# *De Novo* Genome Sequence Assemblies of *Gossypium raimondii* and *Gossypium turneri*

**DOI:** 10.1534/g3.119.400392

**Published:** 2019-08-28

**Authors:** Joshua A. Udall, Evan Long, Chris Hanson, Daojun Yuan, Thiruvarangan Ramaraj, Justin L. Conover, Lei Gong, Mark A. Arick, Corrinne E. Grover, Daniel G. Peterson, Jonathan F. Wendel

**Affiliations:** * USDA/Agricultural Research Service, Crop Germplasm Research Unit, College Station, TX 77845,; † Plant and Wildlife Science Dept. Brigham Young University, Provo, UT 84042,; ‡ Ecology, Evolution, and Organismal Biology Dept., Iowa State University, Ames, IA 50010,; § School of Computing, DePaul University, Chicago, IL 60604,; ** Key Laboratory of Molecular Epigenetics of the Ministry of Education, Northeast Normal University, Changchun, China, and; †† Institute for Genomics, Biocomputing & Biotechnology, Mississippi State University, Mississippi State, Mississippi 39762

**Keywords:** *Gossypium raimondii*, *Gossypium turneri*, cotton, genome sequence, PacBio

## Abstract

Cotton is an agriculturally important crop. Because of its importance, a genome sequence of a diploid cotton species (*Gossypium raimondii*, D-genome) was first assembled using Sanger sequencing data in 2012. Improvements to DNA sequencing technology have improved accuracy and correctness of assembled genome sequences. Here we report a new *de novo* genome assembly of *G. raimondii* and its close relative *G. turneri*. The two genomes were assembled to a chromosome level using PacBio long-read technology, HiC, and Bionano optical mapping. This report corrects some minor assembly errors found in the Sanger assembly of *G. raimondii*. We also compare the genome sequences of these two species for gene composition, repetitive element composition, and collinearity. Most of the identified structural rearrangements between these two species are due to intra-chromosomal inversions. More inversions were found in the *G. turneri* genome sequence than the *G. raimondii* genome sequence. These findings and updates to the D-genome sequence will improve accuracy and translation of genomics to cotton breeding and genetics.

In 2012, the first reference quality cotton genome was brought to fruition through a monumental, collaborative effort using a combination of next-generation sequencing technologies and targeted Sanger sequencing (Paterson *et al.* 2012). *Gossypium raimondii*, a Mesoamerican diploid species, was selected to represent the cotton genus for its small genome size and its relationship to the domesticated polyploid species (Chen *et al.* 2007). Subsequently, this genome has been widely used by the cotton research community, garnering ∼500 citations from a wide spectrum of research. While this genome has been a reliable resource for over 7 years, increased read lengths have improved scaffolding and assembly quality, while development of chromosome conformation capture (3C) techniques have allowed association of sequences within the interphase nucleus but separated by thousands or millions of base pairs along the linear DNA strand (de Wit and de Laat 2012; Peterson and Arick 2018).

The justification for the original *G. raimondii* sequence, *i.e.*, its phylogenetic relatedness to the domesticated allopolyploid species and the recruitment of genetic factors from that subgenome during domestication, make *G. raimondii* and its close relatives potential genetic sources for cotton breeding. *Gossypium turneri* is a species from Sonora, Mexico (Fryxell 1978), that is closely related to *G. raimondii* (Guo *et al.* 2007). Like *G. raimondii*, fiber from *G. turneri* is unspinnable; however, *G*. *turneri* has phenotypic characters with agronomic potential, *e.g.*, caducous bracts, insect resistance, and abiotic stress tolerance (Chen *et al.* 2018).The two species are generally similar, both having a haploid complement of 13 chromosomes and relatively small genome sizes (910 Mb *vs.* 880 Mb in *G. turneri* and *G*. *raimondii*, respectively; (Hendrix and Stewart 2005)). The two species, however, are genetically distinct, as long recognized by taxonomists and their extreme allopatry (*G. raimondii* is from Peru, *G. turneri* from Baja California), as well as by genetic and phylogenetic data (Ulloa *et al.* 2013), (Grover *et al.* 2019). Notably, a previously published draft genome suggests that gene gain and loss may be elevated in *G. turneri* (Grover *et al.* 2019).

Here we describe two *de novo* genome sequences, for *G. raimondii* (D5) and *G. turneri* (D10), which were assembled using newly generated PacBio, Hi-C, and Bionano (*G. raimondii* only) technologies. The *G. raimondii* genome sequence reported here represents an independent effort and identifies three significant assembly errors in the initial publication of *G*. *raimondii*, including a large assembly artifact on the original chromosome 1. We also report a high-quality sequence for *G*. *turneri* that is suitable for various comparative, genetic, and genomic analyses. Together, these genomes represent a useful resource for cotton breeding and for comparative genomics in general.

## Methods & Materials

### Plant material and sequencing

Leaf tissue of mature *G. raimondii* (accession D5-4) and *G. turneri* (accession D10-3) plants was collected at the Brigham Young University (BYU) greenhouse. DNA was extracted using CTAB techniques (Kidwell and Osborn 1992). DNA concentration was measured by a Qubit Fluorometer (ThermoFisher, Inc.). The sequencing library was constructed according to PacBio recommendations at the BYU DNA Sequencing Center (DNASC). Fragments >18 kb were selected for sequencing via BluePippen (Sage Science, LLC). Prior to sequencing, the size distribution of fragments in the libraries was evaluated using a Fragment Analyzer (Advanced Analytical Technologies, Inc). Eight and eleven PacBio cells were sequenced from a single library each for *G. raimondii* and *G. turneri*, respectively, on the Pacific Biosciences Sequel system. For both genomes, the raw PacBio sequencing reads were assembled using Canu V1.6 using default parameters (Koren *et al.* 2017).

HiC libraries were constructed from *G. raimondii* leaf tissue at NorthEast Normal University, China. Sequencing was performed at Annoroad Gene Technology Co., Ltd (Beijing, China). The HiC data of *G. raimondii* was mapped to the previous genome sequence of *G. raimondii* using HiC-Pro (Servant *et al.* 2015), and to the newly assembled CANU contigs of *G. raimondii* PacBio reads by PhaseGenomics. The HiC interactions were used as evidence for contig proximity and in scaffolding contig sequences. An initial draft genome sequence of pseudochromosomes (PGA assembly) was created using a custom python script from PhaseGenomics.

DNA was also extracted from young *G. raimondii* leaves following the Bionano Plant protocol for high-molecular weight DNA. DNA was purified, nicked, labeled, and repaired according to Bionano standard operating procedures for the Irys platform. Two optical maps of different enzymes (*Bsp*QI and *Bss*SI) were assembled using the IrysSolve pipeline on the BYU Fulton SuperComputing cluster. The optical maps were combined into a two-enzyme composite optical map and it was aligned to the PGA assembly using an *in silico* labeled reference sequence. Conflicts between the Bionano maps and the PGA assembly were manually identified in the Bionano Access software by comparing the mapped Bionano contigs to the CANU contigs along the draft genome sequence. Conflicts between datasets were resolved by repositioning and reorienting CANU contigs in PGA ordering files followed by reconstruction of the fasta sequence, provided there was supporting or no-conflict evidence from the optical map ((Durand *et al.* 2016), Supp. Figure 1). Multiple iterations of mapping, conflict resolution, and draft sequence construction resulted in the final, new genome sequence of *G. raimondii*.

Leaf tissue of *G. turneri* was shipped to DoveTail Genomics for DNA extraction and construction of HiC sequencing libraries. These HiC sequencing libraries were sequenced on the Illumina HiSeq 2500 (PE125 bp) at the BYU DNASC. Reads were mapped to the *G*. *raimondii* (Paterson *et al.* 2012) reference genome, and a scaffolded assembly was created for *G. turneri* by Dovetail Genomics. Whole genome alignments identified *in-silico* assembly errors where a contiguous 25.7 Mb of Chromosome 9 (D10_09) was initially placed on D10_12, and the remainder of that chromosome was in smaller scaffolded pieces. Similar to the process above, manual iterations of scaffolding correctly assembled D10_09 and D10_11 using Juicebox (Durand *et al.* 2016). The final genome sequence of *G. turneri* was constructed using a custom python script developed by PhaseGenomics, LLC and consists of 13 assembled chromosomes.

### Repeats and gene annotation

Repeats were identified using a combination of RepeatMasker (Smit *et al.*) and “One code to find them all” (Bailly-Bechet *et al.* 2014), the latter used to assemble multiple adjacent RepeatMasker hits into complete transposable element (TE) copies. RepeatMasker was run for each genome with a custom library, which combines Repbase 23.04 repeats (Bao *et al.* 2015) with cotton-specific repeats. Default parameters were run, except the run was “sensitive” and was set to mask only TEs (no low-complexity). Parameters are available at https://github.com/Wendellab/D5D10. “One code to find them all” was used to aggregate multiple hits into TE models using default parameters. The resulting output was aggregated and summarized in R/3.4.4 (R Development Core Team 2008) using *dplyr* /0.7.4 (Wickham *et al.* 2019). All code can be found at https://github.com/Wendellab/D5D10.

The MAKER-P pipeline (Cantarel *et al.* 2008) was used to annotate *G. raimondii* and *G. turneri* genomes after masking repetitive elements with RepeatMasker (Smit *et al.*) using a custom database that enriched for cotton-specific repeat sequences.

*Gossypium raimondii* was annotated using the iterative MAKER-P method previously described (Grover *et al.* 2017) with the following modifications: (1) assembly of RNA-seq data using Mikado (Venturini *et al.* 2018); (2) RNA-seq assembly provided as another prediction source instead of ESTs evidence; and (3) updated software versions. The raw RNA-seq reads are available from the SRA (PRJNA493521). The assembly and annotation quality for each genome was validated via the BUSCO (Simão *et al.* 2015) pipeline, which evaluates completeness by characterizing the presence, fragmentation, and/or duplication of highly conserved genes. Single-copy syntenic orthologs were inferred using MCScanX (Wang *et al.* 2012) with a minimum of 50 genes in a syntenic block and gap penalty of 2. Any gene belonging to two different syntenic groups was removed.

### Data availability

The assembled genome sequences of *G. raimondii* (PRJNA493304) and *G. turneri* (PRJNA493521) are available in NCBI (CP032553-CP032565 and CP032571-CP032583, respectively). The raw data for *G. raimondii* and *G. turneri* are also available in NCBI (SRR6356446 and SRR7957402, respectively). Supplemental material available at FigShare: https://doi.org/10.25387/g3.9702299.

## Results and Discussion

### Genome assemblies

We report two *de novo* genome sequences for the genus *Gossypium*, a new and corrected assembly for *G. raimondii* (D5) and a new reference-quality assembly for the closely related *G. turneri* (D10). These new genomes integrate multiple sequencing technologies and provide a more accurate representation of each cotton genome. Notwithstanding the utility of the original *G. raimondii* sequence (Paterson *et al.* 2012), it has become evident that the genome sequence contained minor assembly errors.Our genome sequence reported here provides an improved *G. raimondii* assembly using PacBio long read sequencing technology and corrects some errors in the genome sequence that have been identified (Du *et al.* 2018; Wang *et al.* 2019).

The *G. raimondii* genome was assembled from 43.7x PacBio coverage of raw sequence reads. The assembly consisted of 187 contigs with an N50 of 6.3Mb (Table 1). The contigs were scaffolded using HiC by PhaseGenomics and the pseudomolecules were manually adjusted using JuiceBox (Durand *et al.* 2016). The final scaffolded assembly was independently verified using a composite optical map of two different enzymes. A comparison of assembly metrics between the previous genome sequence and our new genome sequence of *G. raimondii* illustrates a 45x improvement in contig length and a 97x reduction in the number of gaps. The cumulative gap length of the new assembly (17.6 kb) was reduced by 647x compared to the assembled gaps of the previous genome sequence (11,391 kb). The final genome assembly size was 14.9 Mb smaller than the previous assembly, representing 98% of previously assembled genome sequence in length.

**Table 1:** Assembly metrics of the *G. turneri* genome, the *G. raimondii* (our current assembly, D5), and the previous *G. raimondii* assembly (Paterson *et al.* 2012)

	*G. turneri* (D10)	*G. raimondii* (D5)	*G. raimondii* (2012)
Contigs	220	187	16,924
Max Contig	23,475,487	24,216,129	1,162,971
Mean Contig	3,432,648	3,929,767	43,597
Contig N50	7,909,293	6,291,832	136,998
Contig N90	1,624,019	2,044,991	32,166
Total Contig Length	755,182,540	734,866,495	737,837,083
Assembly GC	33.21	33.19	33.19
Scaffolds	13	13	13
Max Scaffold	67,704,245	65,701,939	70,713,020
Mean Scaffold	58,092,557	56,529,546	57,632,930
Scaffold N50	60,464,062	58,819,159	62,175,169
Scaffold N90	50,570,303	46,322,098	45,765,648
Total Scaffold Length	755,203,240	734,884,094	749,228,090
Captured Gaps	207	174	16,911
Max Gap	100	200	63,138
Mean Gap	100	101	674
Gap N50	100	100	2,607
Total Gap Length	20,700	17,599	11,391,007

This is the first *de novo* genome sequence for *G. turneri*. The *G. turneri* genome was assembled from 73.2x PacBio of raw sequence reads. The assembly consisted of 220 contigs with an N50 of 7.9Mb (Table 1). Similar to the *G. raimondii* sequence, these contigs were scaffolded by Dovetail Genomics and the pseudomolecules were manually adjusted using JuiceBox. Bionano data were not collected for *G. turneri*. The *G. raimondii* Bionano data were uninformative when aligned to the *G. turneri* genome sequence (because the distances between labeled recognition sites were too different). After creation of the sequence assembly, the *G. raimondii* HiC sequence reads were also mapped to the *G. turneri* genome sequence (and *vice versa*). While the number of mapped reads was reduced significantly (29.90% and 12.67%, respectively), there were no association anomalies detected between genomes.

**Figure 1:**
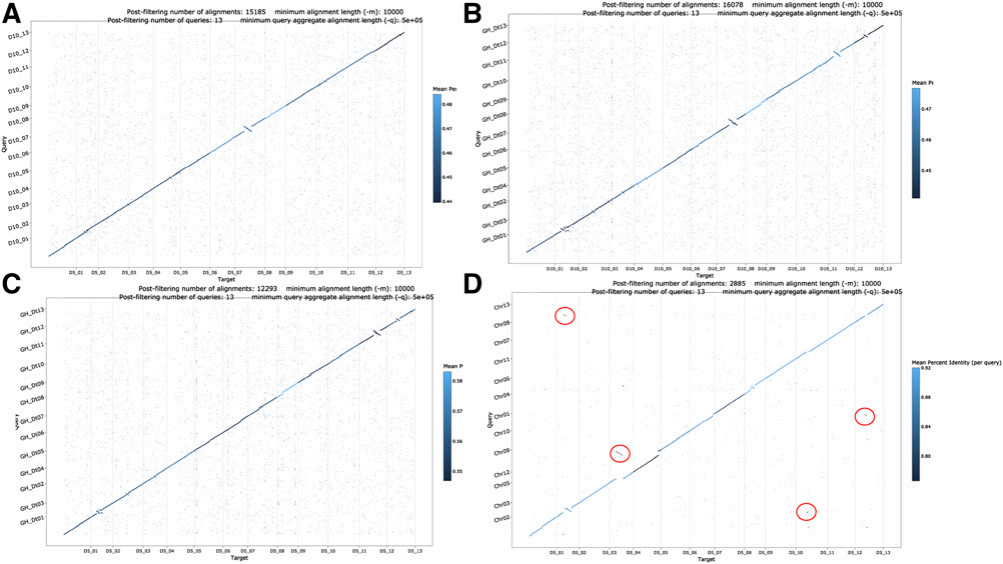
Genome comparisons between *G. raimondii* (D5), *G. turneri* (D10), *G. raimondii* (2012), and the D_T_-genome of *G. hirsutum* (D_T_). A) Genome alignment between *G. turneri* (D10) and *G. raimondii* (D5). B) Genome alignment between D_T_ and D10. C) Genome alignment between D_T_ and D5. D) Genome alignment between D5 (2012) and D5 (new). Red circles indicate assembly errors in the 2012 sequence as identified by these alignments and independent HiC data (*e.g.*, D5_13 – Chr01, D5_11 – Chr03, D5_04 – Chr09, D5_02 – Chr13).

The assembled genome sequences were also verified by alignments to the D_T_-genome of *G. hirsutum* (Wang *et al.* 2019) and to the previous genome assembly of *G. raimondii* (Figure 1). The chromosomes had general agreement in their alignments between the four independently assembled sequences (old and new *G. raimondii*; *G. turneri*; D_T_ of *G. hirsutum*). Such colinearity was also previously identified between cotton genomes. For example, genetic maps of *G. hirsutum* (*e.g.*, (Byers *et al.* 2012)) were used to previously verify and sometimes establish proper scaffolding between contigs (Paterson *et al.* 2012).

### Error Correction in G. raimondii genome sequence

**Figure 2:**
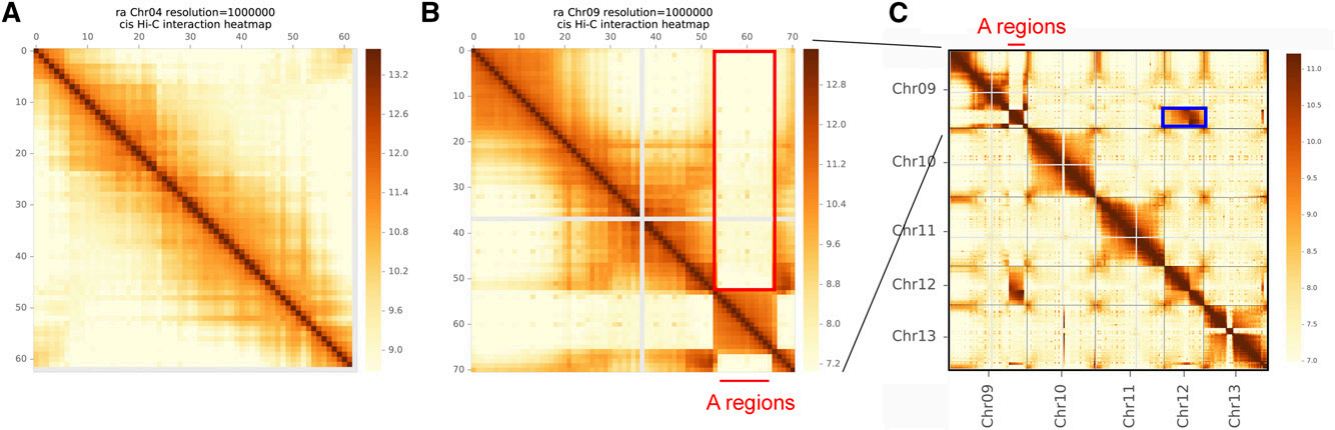
HiC interactions detected in the previously published *G. raimondii* genome sequence (Paterson *et al.* 2012). A) Most interaction maps of chromosome sequences suggested that the genome sequence was assembled in the correct order. B) A sequence was incorrectly assembled within Chr. 9 (now D5_05) that created a large insertion (red box). Few interactions were found between the inserted segment and the remainder of Chr. 9. C) Corresponding interactions were identified in the HiC interaction plot between Chr. 9 and Chr. 12 (now D5_04), as well as ‘pinch’ within the diagonal interaction map in Chr. 12, indicating the true position of the incorrectly assembled sequence.

Errors were identified in the previous *G. raimondii* sequence (Paterson *et al.* 2012). In the previous genome sequence, the chromosomes were named to be consistent with previous genetic maps; however, a new chromosome naming convention has been used for diploid and allotetraploid cotton (Li *et al.* 2015; Zhang *et al.* 2015; Du *et al.* 2018), where homeologous chromosomes are organized in sequence pairs (*e.g.*, A_T__01 - A_T__13 [Chr. 01 - Chr. 13] are homeologs of D_T__01 - D_T__13 [Chr. 14 – Chr. 26], respectively). We have adopted this new naming convention for the homologous chromosomes of these two genomes. Structural errors in the previously published sequence were identified by genome alignments (Figure 1) and by mapping HiC reads to the genome sequence (Figure 2, Supp Figure 1). The largest error was an assembly-derived translocation of D5_04 (previously Chr. 12) on D5_05 (previously Chr. 09) (Figure 2). Additional, smaller errors were found between Chr. 01 (now D5_07) and Chr. 13 (now D5_13); Chr. 02 (now D5_01) and Chr. 13 (now D5_13); Chr. 03 (now D5_02) and Chr. 13 (now D5_13); Chr. 02 (now D5_01) and Chr. 03 (now D5_02); Chr. 02 (now D5_01) and Chr. 07 (now D5_11); and Chr. 03 (now D5_02) and Chr. 07 (now D5_11) (Supp Figure 1). These corrections based on alignment and HiC data were also supported by the alignment of Bionano data.

**Figure 3:**
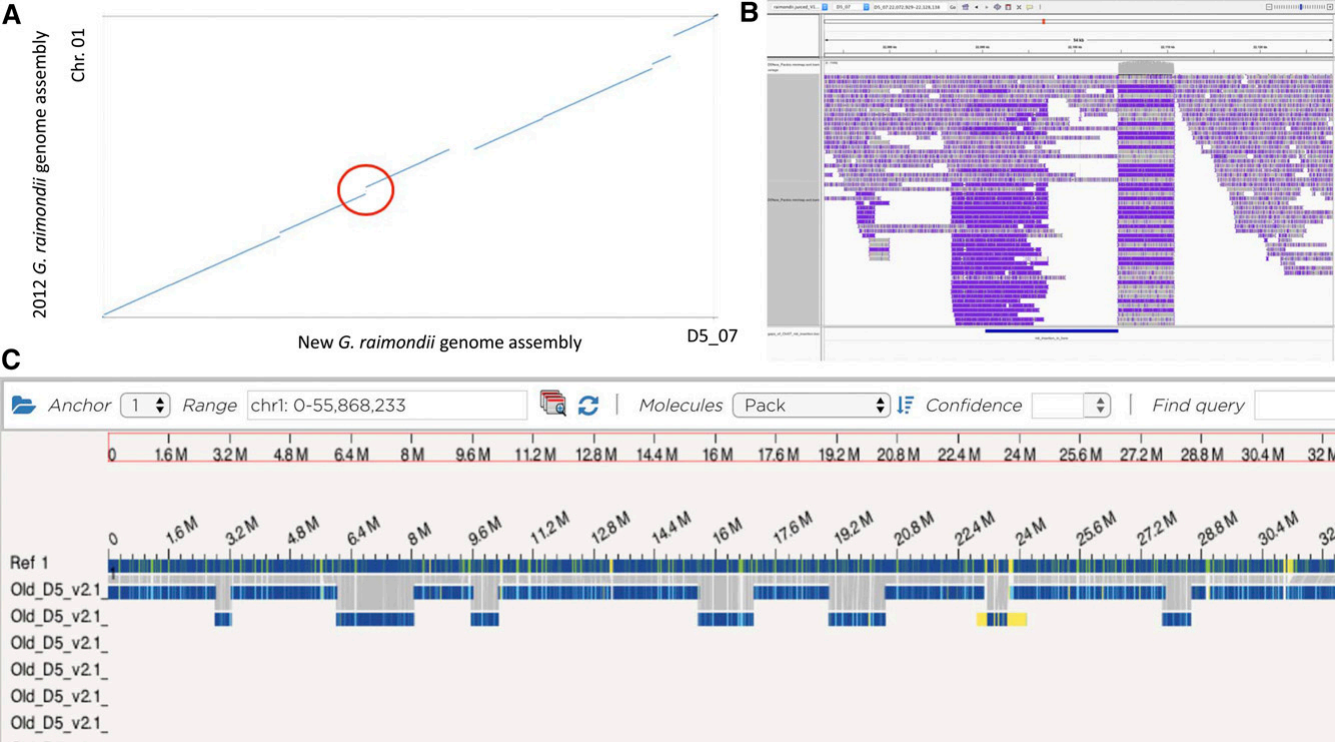
Genomic assembly data of the new *G. raimondii* sequence suggest that the previously reported mitochondrial insertion was likely due to an assembly error. A) Genome alignments between *G. raimondii* Chr. 01 (Paterson *et al.* 2012) and our new genome sequence of D5_07. The red circle indicates the putative position of the mitochondrial genome insertion in the previous *G. raimondii* sequence relative to the new assembly. B) Alignment of *G. raimondii* PacBio reads (Track 2) to the new reference genome of *G. raimondii* (Track 1). The multi-colored bars represent individual PacBio reads (Track 2). The previous reference genome of *G. raimondii* had a mitochondrial insertion somewhere in this 14kb region indicated by the blue bar of Track 3. There are no PacBio reads that span the gap between the flanking regions of the 6,071 repeat and the repeat itself. C) Bionano data mapped to the previous reference genome sequence of *G. raimondii* (Paterson *et al.* 2012) also suggest an insertion of a sequence that is non-contiguous in the flanking regions. The Ref1 track reference to the originally published genome sequence of *G. raimondii* with a mitochondrial insertion between ∼23Mb and ∼24Mb. Independently constructed Bionano contigs were aligned to the 2012 reference sequence. A Bionano contig matched the reference sequence in the mitochondria insertion region, but the flanking regions of the Bionano contig (yellow) did not match flanking Bionano contigs or the reference sequence.

We also inspected a reported nuclear mitochondrial genome insertion (NUMT) on D5_07 (previously Chr. 1, Figure 3) located between coordinates 23.1Mb and 25Mb (Paterson *et al.* 2012). This region appears to have been the result of assembly error. Alignment of the two genomes (previous D5 genome *vs.* new D5 genome) identified a 1.26Mb segment that was inserted into the old sequence and not found in our new *de novo* assembly. Bionano data also indicated an insertion in the old assembly while the ‘inserted’ Bionano contig was unmapped in the new assembly of D5 (Figure 3C).

Since NUMTs evolve more quickly than do functional mitochondrial gene sequences, we also inspected the sequence similarity of the NUMT to the mitochondrial genome sequence of *G. raimondii* (Chen *et al.* 2017). The NUMT exhibited high similarity to the published *G. raimondii* mitochondrial genome (99.8% PID over 94% of region between Chr01:23,100,000-25,000,000). On an individual gene basis, over half of the genes contained within the putative NUMT were over 99% identical to the published sequence in the *G. raimondii* mitochondrial genome, with an average of 95% similarity. Considering the D-genome alignments and Bionano data presented above, the NUMT was more likely an assembly artifact than a recent insertion event in the *G. raimondii* genome.

### Structural Variations Between the D-genomes

Comparisons between *G. raimondii* and *G. turneri* revealed several structural differences between the two genomes (Supp Figure 2). The genomes were largely colinear and no significant duplicated segments (relative to the genome alignments) were found in either genome (Figure 1**)**. The assembled sequence of the *G. turneri* genome was 20.3 Mb longer than the *G. raimondii* genome and the gene content was similar (see below). The largest number of structural variants between the two genomes were chromosomal inversions. We identified several relative inversions between the two *de novo* genomes (Table 2). Inversions were manually identified in the genome alignment output file. A total of 64 Mb genome sequence had an inverted order between these two genomes.These regions included a total of 2,592 genes (∼6.4% of gene total number). The largest structural variant was an inversion on D10_08 (Figure 1, Supp Figure 3 - 15). This inversion could have been the result of misassembly, but the putative break points had clear overlapping, individual PacBio reads in *G. raimondii* (Supp Figures 6 & 9) and in *G. turneri* (Supp Figures 10 & 13**)**. In addition, both genomes had consistent HiC patterns for the D8 chromosome where an inversion of ∼16 Mb would have been clearly identified had it been the result of assembly error in one of the two genome sequences (Supp Figure 1).

**Table 2:** Inversions between the *de novo* genome assemblies of *G. turneri* and *G. raimondii*

Chromosome	Inv. number	Total Length	Gene number
1	9	4,856,224	132
2	9	7,086,444	114
3	5	5,569,613	431
4	4	2,192,874	60
5	5	4,213,508	179
6	6	1,597,287	164
7	3	2,453,735	159
8	7	16,167,439	345
9	8	5,741,456	267
10	2	417,545	9
11	9	7,708,678	501
12	4	1,771,113	44
13	10	4,944,400	187
Total	81	64,720,316	2592

We also compared the *G. turneri* and *G. raimondii* genome sequence to other *Gossypium* genomes (Supp Figure 16 & 17, (Du *et al.* 2018; Wang *et al.* 2019)). If a large inversion between *G. turneri* and *G. raimondii* was 1) also present in the genome alignment between *G. arboreum* and *G. turneri* and 2) was not present in the genome alignment between *G. raimondii* and *G. arboreum* then it was considered as an inversion derived during the natural evolutionary history of the *G. turneri* genome (similar logic for inversions derived in *G. raimondii* or *G. arboreum*). The inversions need to be large (>2 Mb) and present in only one genome to be confident about its description without further investigation. The largest inversions on chromosomes D10_03, D10_05, D10_07, and D10_08 appear to be specific to *G. turneri* (36% of the length of total inversions). Chromosome rearrangements (inversions and other events) specific for *G. arboreum* were found on A2_01, A2_02, A2_03, A2_07, and A2_11. Only one inversion (D5_13, 2.6 Mb) was found to be specific to the *G. raimondii* genome. Perhaps, these inversions were part of the speciation process between the different *Gossypium* genomes.

### Gene annotations

Similar numbers of genes were found in the annotation of each genome. Annotation of the genomes of *G. turneri* and *G. raimondii* identified 38,489 and 40,743 gene models respectively (Table 3). BUSCO analysis reported >90% completeness scores for both *G. turneri* and *G. raimondii* genome assemblies, indicating that the evolutionarily-conserved core gene set was present in both *de novo* assemblies (Supp. Figure 18). Using MCScanX, we were able to identify 23,499 syntenic orthologs shared between the two species, indicating that the gene order and gene compliment are largely conserved between these two species. Genes in 34 of these syntenic orthologs were inferred to have more than one syntenic ortholog; these genes were removed from the dataset, resulting in 23,465 high-confidence syntenic orthologs (Supp. File 1). While not every gene was categorized into syntenic relationships, this is not surprising given that genes present in tandem arrays were excluded from this analysis (a default setting of MCScanX), gene loss has likely occurred in both species since they last shared a common ancestor, and subtle differences in gene annotation in the two genome assemblies likely lead to slight differences in overall gene content.

**Table 3:** Each of the *de novo* genome assemblies were annotated for gene content using Maker-P

Predicted Features	*G. turneri* (D10)	*G. raimondii* (D5)	*G. raimondii* (2012)
CDS	205,333	235,836	486,043
exon	200,384	236,559	527,563
gene	38,489	40,743	37,505
mRNA	39,553	41,030	77,267

### Repeats

Transposable element content was predicted for both *de novo* genomes and compared to the existing *G. raimondii* reference sequence (Paterson *et al.* 2012). As expected, the *de novo G. raimondii* genome had nearly identical predicted TE content with the previous *G. raimondii* genome sequence (Table 4). This difference is not significant and can be attributed to slight differences in assembly of repetitive regions. Consistent with the larger size of *G. turneri* than *G. raimondii* (910 Mb *vs.* 880 Mb), the *G. turneri* genome assembled an additional 8.5 Mb and 10.6 Mb of repetitive sequence, relative to the previous and new *de novo G. raimondii* genome sequences, respectively. Generally, the *G. turneri* genome sequence has slightly fewer DNA TEs and more LTR retrotransposons than the two *G. raimondii* genomes, both with respect to absolute content and percent of genome (Table 4). No non-LTR retrotransposons (*e.g.*, LINE/SINE) were detected. For all three genome assemblies, retrotransposons comprise approximately 36% of the genome sequence, whereas all DNA elements combined comprise just under 3% in each. These results are consistent with a previous analysis of low-coverage sequencing results of these two genomes (Grover *et al.* 2019).

**Table 4:** Repetitive content of the newly sequenced *G. turneri* and *G. raimondii* genomes, and the previously published *G. raimondii* (Paterson *et al.* 2012). No LINE or SINE elements were detected. The genome size of *G. turneri* is 910 Mb and *G. raimondii* is 880 Mb

	*G. turneri*			*G. raimondii*			*G. raimondii* (Paterson *et al.* 2012)		
Family	Fragments	Copies	Total (Mb)	Fragments	Copies	Total (Mb)	Fragments	Copies	Total (Mb)
**DNA**	20,199	12,453	18.28	22,503	13,764	20.63	23,474	13,969	20.27
***CMC/EnSpm***	2	1	0.00	2	2	0.00	14	9	0.00
***EnSpm/CACTA***	2,443	1,385	3.92	3,172	1,864	5.24	3,648	1,878	4.87
***Harbinger***	30	22	0.01	58	41	0.03	42	28	0.02
***hAT***	2,725	1,712	1.01	3,079	1,895	1.01	3,209	1,966	1.03
***L1***	1,255	638	1.56	1,256	618	1.49	1,290	633	1.54
***Mariner/Tc1***	98	51	0.07	76	40	0.06	84	43	0.06
***MuDR***	13,590	8,592	11.71	14,828	9,280	12.79	15,145	9,381	12.73
***MULE-MuDR***	52	50	0.01	21	19	0.00	25	23	0.00
***PIF-Harbinger***	4	2	0.00	11	5	0.00	17	8	0.01
**LTR**	338,644	199,672	277.72	325,760	190,122	264.75	336,908	196,564	267.24
***LTR***	224	216	0.02	214	206	0.02	311	304	0.03
***Copia***	48,098	28,294	45.51	48,911	29,032	45.29	50,993	29,965	45.72
***Gypsy***	290,322	171,162	232.19	276,635	160,884	219.44	285,604	166,295	221.49
**Total**	**358,843**	**212,125**	**296.00**	**348,263**	**203,886**	**285.38**	**360,382**	**210,533**	**287.51**

### Conclusion

Genome sequences of many plants have been recently published, and in fact are too numerous to cite here. Many of these previously reported genome sequences are being revisited with long-read technology of PacBio or Oxford Nanopore. In this report, we present new *de novo* genome sequences for *G. raimondii* and *G. turneri* based on PacBio long-read sequence technology. Both of these genomes are closely related to the D_T_-genome of cultivated tetraploid cotton. These sequences provide an evolutionary perspective for comparative genomics of the *Gossypium* clades as well as providing useful resources for the genetic improvement of cotton. Because of the economic relevance of the *Gossypium* genus, additional genome sequences of related *Gossypium* species will continue to be studied and revised in the future.
